# IlsA, A Unique Surface Protein of *Bacillus cereus* Required for Iron Acquisition from Heme, Hemoglobin and Ferritin

**DOI:** 10.1371/journal.ppat.1000675

**Published:** 2009-11-26

**Authors:** Nadine Daou, Christophe Buisson, Michel Gohar, Jasmina Vidic, Hélène Bierne, Mireille Kallassy, Didier Lereclus, Christina Nielsen-LeRoux

**Affiliations:** 1 INRA-UR1249 Génétique Microbienne et Environnement, La Minière, Guyancourt, France; 2 Laboratoire de Biotechnologie, Université Saint-Joseph, Beyrouth, Lebanon; 3 INRA-UR892 Unité de Biologie Physico-Chimique des Prions, Virologie et Immunologie Moléculaires, Jouy en Josas, France; 4 Unité Interaction Bactéries Cellules, Institut Pasteur, INSERM U604 – INRA USC2020, Paris, France; The University of Texas-Houston Medical School, United States of America

## Abstract

The human opportunistic pathogen *Bacillus cereus* belongs to the *B. cereus* group that includes bacteria with a broad host spectrum. The ability of these bacteria to colonize diverse hosts is reliant on the presence of adaptation factors. Previously, an IVET strategy led to the identification of a novel *B. cereus* protein (IlsA, Iron-regulated leucine rich surface protein), which is specifically expressed in the insect host or under iron restrictive conditions *in vitro*. Here, we show that IlsA is localized on the surface of *B. cereus* and hence has the potential to interact with host proteins. We report that *B. cereus* uses hemoglobin, heme and ferritin, but not transferrin and lactoferrin. In addition, affinity tests revealed that IlsA interacts with both hemoglobin and ferritin. Furthermore, IlsA directly binds heme probably through the NEAT domain. Inactivation of *ilsA* drastically decreases the ability of *B. cereus* to grow in the presence of hemoglobin, heme and ferritin, indicating that IlsA is essential for iron acquisition from these iron sources. In addition, the *ilsA* mutant displays a reduction in growth and virulence in an insect model. Hence, our results indicate that IlsA is a key factor within a new iron acquisition system, playing an important role in the general virulence strategy adapted by *B. cereus* to colonize susceptible hosts.

## Author Summary

Iron is an essential compound for almost all living organisms, taking part in basic cellular homeostasis. Preventing access to iron sources for invading pathogens is one of the defense systems used by hosts to avoid pathogen colonization. To counteract this, pathogens have developed mechanisms to acquire nutrient iron during infection. *Bacillus cereus* is an opportunistic bacterium able to infect both insects and mammals; thus, it should have systems enabling iron uptake from these hosts. Here we describe, for the first time, a unique surface protein, called IlsA, which is essential for iron uptake from two very different iron binding molecules: ferritin and hemoglobin. IlsA is only produced in iron limited environments. We show that during insect infection, its expression is specific to insect hemocoel (blood), where ferritin is the major iron-binding molecule. Interestingly, the IlsA mutant has reduced survival in *in vivo* infection and *in vitro* when heme, hemoglobin and ferritin are the sole iron sources available. Thus, as IlsA is important for iron uptake from the major iron rich molecules in insects and mammals, we suggest that this new iron acquisition system may be a key factor that is evolutionary adapted to infection of such diverse hosts.

## Introduction

Iron is an essential element for most organisms, including bacteria, because it is involved in many cellular processes including aerobic respiration, amino acid and nucleotide biosynthesis [1],[2]. Since free iron is highly toxic for the cells, its homeostasis is strictly regulated in living organisms. Protection against iron is achieved by iron sequestration in carrier proteins such as transferrin, lactoferrin, ferritin or as iron-binding to the heme in hemoproteins. Thus, the lack of free iron is an obstacle that bacteria must overcome, when invading a host. In order to scavenge iron from the host iron-binding proteins, bacteria have developed two principal high affinity iron-uptake systems, which are considered to be important virulence factors. One system is based on the secretion of siderophores that capture iron from iron-binding proteins by the virtue of a superior binding strength. The siderophores are then recognized by specific membrane anchored binding proteins and internalized into the cytosol [3],[4]. The second system involves direct binding to host iron rich proteins via specific bacterial surface receptors which subsequently interact with membrane bound ABC transporters and permeases allowing iron transfer into the cytosol. These systems have been more studied in Gram-negative compared to Gram-positive bacteria [5]–[8]. The majority of these iron-uptake systems are under the control of the repressor Fur (Ferric uptake regulator) [3]. In Gram-positive bacteria, the best characterized system relying on bacterial surface proteins is the iron-regulated surface determinants (Isd) of *Staphylococcus aureus*. The Isd system implements cell wall proteins that act as hemoprotein receptors [9],[10] because of the presence of several copies of the conserved NEAT domains (for NEAr iron Transport), which play a key role in heme and hemoproteins binding and transport [11],[12]. An Isd system has been also studied in *Bacillus anthracis*. In addition to cell wall proteins, the *B. anthracis* Isd system uses secreted proteins that contain NEAT domains which act as hemophores, enabling heme acquisition from hemoglobin [13],[14].

The Gram-positive, spore-forming and human opportunistic pathogen, *Bacillus cereus*, belongs to the *Bacillus cereus* group, which also includes the entomopathogen, *Bacillus thuringiensis*, and the etiological agent of anthrax in mammals, *B. anthracis*. These three closely related species share a large number of chromosomal determinants, whereas their host-specific toxins are carried on plasmids [15]–[17]. *B. cereus* is generally associated with human food poisoning, resulting from the diarrhea and the emetic toxins [18]. However, *B. cereus* can also cause serious infections such as endophthalmitis, pneumonia and meningitis [19]–[21]. In addition, a new *B. cereus* species was found to cause severe respiratory illness resembling anthrax [22]. To date, virulence of *B. cereus* has been ascribed to different extracellular factors that are under the control of the pleiotropic regulator PlcR, which is part of a quorum sensing system [23],[24]. The PlcR regulon is important for virulence in both mice (intranasal infection) and insects [25]. However, other factors are implicated in the pathogenesis of *B. cereus*. Indeed, little research has been carried out on virulence genes involved in the adaptation and the persistence of *B. cereus* in the host and specifically on genes involved in iron acquisition.

Previous studies have shown that *B. cereus* secretes two siderophores: Bacillibactin and Petrobactin [26]. A recent study has identified a surface binding receptor protein as a potential receptor to these siderophores [27]. The ability of *B. cereus*, to acquire iron from host iron-rich sources, has received little attention; two independent research groups have reported contradictory results on the capacity of *B. cereus* to use transferrin [28],[29]. In addition, other studies have reported that *B. cereus* can use hemoglobin, hemin and other hemoproteins such as hemoglobin-haptoglobin and hemin-albumin for growth [28],[30],[31]. The majority of these studies focused on the identification of iron sources that can be used by *B. cereus*, whereas mechanisms used by *B. cereus* to scavenge iron from the host and especially those involving surface proteins have not yet been reported, except for the recently described siderophore binding protein [27].

**Figure 1:**
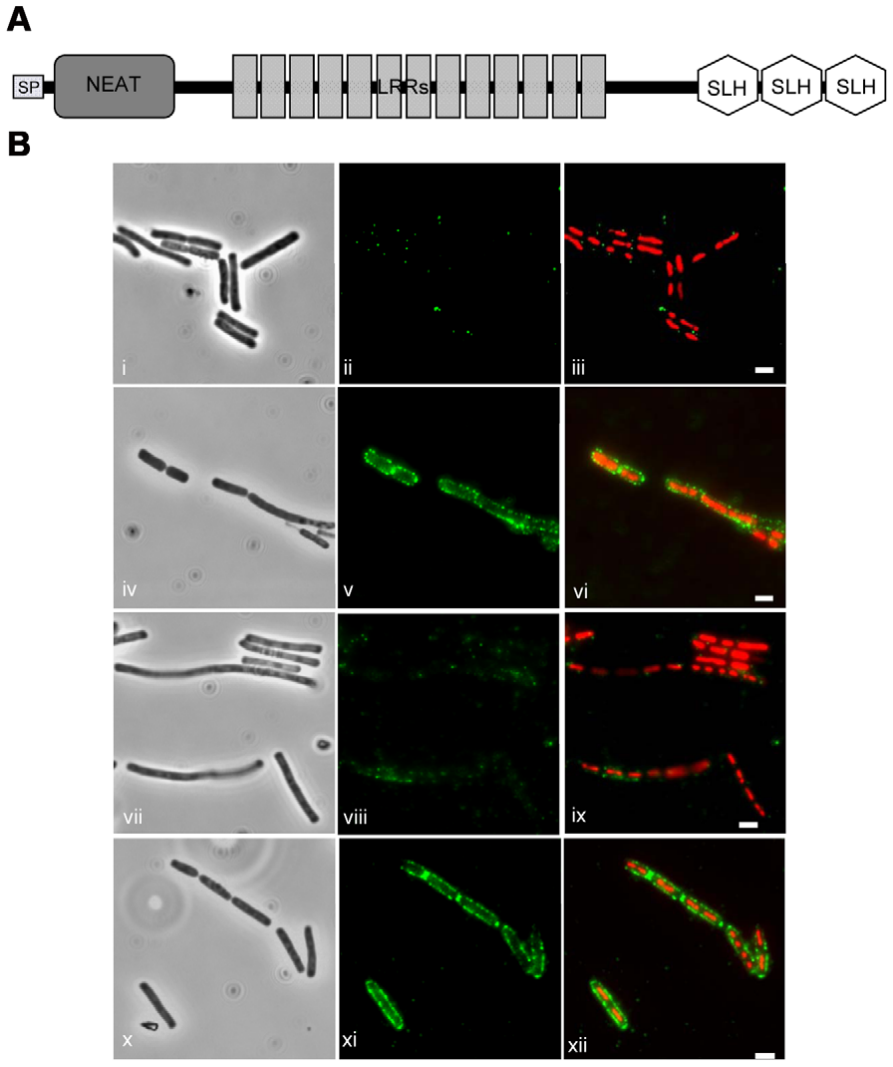
Schematic representation of the protein IlsA and its localization. (A) The predicted conserved domain of the protein IlsA. The N-terminal putative signal peptide (SP) for secretion is indicated by a bold square. The three conserved domains: NEAT (NEAr iron Transporter), LRR (Leucine-Rich Repeat) and SLH (Surface Layer Homology) are indicated. (B) Localization of IlsA on the surface of *B. cereus*. The wild-type grown in LB medium (panels i–iii) and in LB+150 µM 2,2′-dipyridyl medium (panels iv–vi), the mutant Δ*ilsA* grown in LB+150 µM 2,2′-dipyridyl medium (panels vii–ix), and the complemented Δ*ilsA*+pHT304Ω*ilsA* strains grown in LB+150 µM 2,2′-dipyridyl medium (panels x–xii), were analyzed by light microscopy with phase contrast (panels i,iv,vii,x) and immunofluorescence (panels ii,v,viii,xi) using the IlsA polyclonal antibody. The merged images (panels iii,vi,ix,xii) show the DAPI stained DNA in red and IlsA in green. IlsA is localized at the bacterial surface of the wild-type and only in iron depleted condition (panels v,vi). Interruption of *ilsA* abolished the presence of IlsA on the bacterial surface (panels viii,ix) and the complementation of the mutation restored the localisation (panel xi,xii). White bars represent 2 µm.

In our previous study, an IVET strategy (In Vivo Expression Technology) enabled us to identify a *B. cereus* gene specifically expressed in insect larvae infected via the oral route [32]. It was shown that the transcription of this gene was induced in response to iron restrictive conditions. The presence of a Fur-binding box in the gene promoter region and a conserved NEAT domain in the protein are in agreement with an iron-dependent regulation. The presence of a N-terminal peptide signal and SLH domains (S-layer homology) suggest that the protein might be located on the bacterial surface (Figure 1A). This protein was designated IlsA, for Iron regulated leucine-rich surface protein. Moreover, IlsA was involved in the virulence of *B. cereus* in the lepidopteran *Galleria mellonella* larva infected by the oral route [32]. A BLAST search indicates that IlsA-like proteins are restricted to *B. cereus* group bacteria.

Here, we demonstrate that IlsA is a surface protein absolutely required for iron-uptake from several host proteins by direct binding to hemoglobin, hemin and ferritin. This is the first time that a NEAT domain protein has been shown to be involved in iron-uptake from ferritin. Furthermore, *in vivo* studies show that IlsA is specifically expressed in the hemocoel of *G. mellonella* and that the reduced virulence of an *ilsA* mutant correlates with a decrease of its *in vivo* growth capacity. Our results suggest that IlsA is an important adaptation factor required for the development of *B. cereus* in the host. Finally, these findings provide new insights into the ecology of this bacterial group that is able to colonize hosts as diverse as mammals and insects.

## Materials and Methods

### Bacterial stains and growth conditions

*Bacillus cereus* strain ATCC 14579 (laboratory stock) was used throughout this study.

The mutant *B. cereus* ATCC 14579 Δ*ilsA* was previously constructed by allelic exchange through a double cross-over event by introducing a tetracycline resistant cassette [32]. *E. coli* K12 strain TG1 was used as a host for cloning experiments. Dam–, Dcm– *E. coli* strains ET 12567 (laboratory stock) were used to generate unmethylated DNA for electro-transformation in *B. cereus*. For electro-transformation, *B. cereus* was grown in BHI (Brain Heart Infusion, Difco) broth. *E. coli* and *B. cereus* strains were transformed by electroporation as previously described [33],[34]. *E. coli* BL-21 [F^−^, *ompT*, *HsdS* (r_B_^−^, m_B_^−^), *gal*] strain was used for expression and purification of the Glutathione S-transferase (GST) fusion protein (GST-IlsA). *E. coli* and *B. cereus* were cultured in LB (Luria–Bertani) broth, with vigorous shaking (175 rpm) at 37°C. Antibiotics for bacterial selection were used at the following concentrations: ampicillin (100 µg ml^−1^ for *E. coli*), erythromycin (10 µg ml^−1^ for *B. cereus*) and tetracycline (10 µg ml^−1^ for *B. cereus*). The iron chelator, 2,2′-dipyridyl and the iron sources, hemoglobin, hemin, ferritin, transferrin and lactoferrin, were obtained from Sigma-Aldrich, St. Quentin Fallavier, France. The iron sources were used at a concentration (see below) that permits the growth of *B. cereus*. The ferric chloride (FeCl_3_) was used at a final concentration of 0.2 mM. All iron solutions were sterilized by passage through 0.22 µm pore size filter.

### DNA manipulations

Chromosomal DNA was extracted from *B. cereus* cells with the Puregene DNA Purification Kit (Gentra, Minneapolis, USA). Plasmid DNA was extracted from *E. coli* and *B. cereus* using QIAprep spin columns (QIAgen, France). For *B. cereus*, 5 mg ml^−1^ of lysosyme was added and cells were incubated at 37°C for 1 h. Oligonucleotide primers were synthesized by Proligo (Paris, France). PCRs were performed in a thermocycler PTC-100™ (MJ-Research, Inc., USA). Amplified fragments were purified using the QIAquick PCR purification Kit (QIAgen). Restriction enzymes (New England Biolabs, USA) and T4 DNA ligase (Invitrogen, USA) were used as recommended by the manufacturer. Digested DNA fragments were separated by electrophoresis on 1% agarose gels and extracted from gels using the QIAquick gel extraction Kit (QIAgen).

### Complementation of the *ilsA* mutant strain

The genetic complementation of the strain *B. cereus* Δ*ilsA* was carried out as follows. A DNA fragment corresponding to the gene *ilsA* was amplified by PCR using the *B. cereus* ATCC 14579 genomic DNA as a template and the primers *ilsA*-forward (5′-AACTGCAGGGGCTTTTTTATTTTGTACC-3′) and *ilsA*-reverse (5′-CGGAATTC GTGAGGGCTACTAATCAGTTG-3′). The PCR product was digested with EcoRI and Pst1 restriction enzymes and inserted into the plasmid pHT304 by ligation using the T4 DNA ligase [35]. The resulting plasmid (pHT304Ω*ilsA*) was amplified in *E. coli* and then introduced into the mutant strain *B. cereus* Δ*ilsA* by electroporation.

### IlsA purification and antibody production

Glutathione *S*-transferase (GST) and GST-IlsA were purified as recombinant proteins from *E. coli*. The expression plasmid p*gst*-*ilsA* was constructed by PCR amplification of the *ilsA* sequence from the *B. cereus* genome using the primer pair *ilsA-*forward-2 (5′-CGGAATTCTTGAAAAAAAATTATATGAAGG-3′) and *ilsA-*reverse-2 (5′-CCCTCGAGTTATTTCTTTATTGCATTATAC-3′). The DNA fragment was digested with EcoRI/XhoI, cloned into pGEX6P1 (Amersham Biosciences) digested with the same restriction enzymes and transferred into *E. coli* Bl-21 by electroporation. *E. coli* Bl-21 strain harboring pGEX6P1-*ilsA* was grown to log phase in LB medium with ampicillin at 37°C. The expression of the fusion protein GST-IlsA was induced by adding IPTG (isopropyl-β-d-thiogalactopyranoside) to a final concentration of 1 mM and the cultures were incubated for 3 hours at 30°C. The bacteria were collected following centrifugation at 7700 rpm for 15 min in 50 ml tubes and suspended in 1X PBS containing 1% Triton X-100. Bacteria were lysed on ice by sonication using a Branson sonicator 250. Bacterial lysate was centrifuged at 7600 rpm for 15 min at 4°C, and the pellet containing the fusion protein IlsA-GST was solubilized in 50 mM Tris (pH 8.0) containing 8 M urea. After solubilization in 8 M urea overnight at 4°C, the insoluble pellet was removed by centrifugation at 13000 rpm for 20 min at 4°C. The supernatant containing the solubilized protein was dialyzed against buffer containing Tris 50 mM (pH 8.0) and 1 M urea, for about 5 hours with stirring at 4°C to remove the residual detergent. The solubilized and dialyzed fusion protein was loaded onto a glutathione Sepharose 4B column equilibrated with 1X PBS. After binding the fusion protein, the column was washed with PBS 1X and the target protein GST-IlsA was eluted with 10 mM reduced glutathione.

Purified GST-IlsA was then dialyzed against the PreScission protease buffer (50 mM Tris HCl pH 7.0, 150 mM NaCl, 1 mM EDTA, 1 mM DTT) at 4°C overnight. The GST was then cleaved using the PreScission protease at a concentration of 2 Units µl^−1^ for 4 hours at 5°C. After digestion, the mix was separated on a 10% gel SDS PAGE and the IlsA protein band was cut from the gel and used to raise anti-IlsA antibodies (Proteogenix, Oberhausbergen, France). Purification of IlsA was monitored by SDS PAGE electrophoresis (Figure S1). The GST was prepared using the strain *E. coli* Bl21 harbouring the plasmid pGEX6P1. After induction using IPTG, the bacteria were lysed by sonication and GST present in the cytosol of the bacteria was purified using the glutathione Sepharose 4B column as described above (Figure S1).

### Immunofluorescence analysis

For the localization of IlsA at the bacterial surface, overnight cultures of *B. cereus* wild-type, *B. cereus* Δ*ilsA* and the complemented strain *B. cereus* Δ*ilsA* pHT304Ω*ilsA* were grown in LB medium or in LB medium treated with 150 µM 2,2′-dipyridyl at 37°C and used immediately. Bacteria from stationary phase culture (10^9^) concentrated in 50 µl, were washed twice in phosphate buffered saline (PBS 1X) and fixed with 4% paraformaldhehyde dissolved in PBS 1X on a cover slip. After fixation, bacteria were washed in PBS and then labelled with an anti-IlsA polyclonal antibody diluted at 1∶500 in 1% BSA, followed by an anti-rabbit secondary antibody Alexa 488-conjugated at a dilution of 1∶500 in 1% BSA. Bacterial DNA was also labelled with DAPI at a dilution of 1∶300 in 1% BSA. Labelled bacteria were then sticked with Mowiol to a glass slide and dried at 37°C for 30 min. Preparations were examined under a Zeiss Axiovert 135 microscope. Image acquisition from the Zeiss microscope was carried out with a cooled charge-coupled device camera (Princeton), and the images were processed with Metamorph software (Universal Imaging Corporation).

### Growth assay

*B. cereus* cultures were grown overnight under low iron conditions by inoculating strains in LB medium supplemented with 200 µM 2,2′-dipyridyl. Overnight cultures were centrifuged and washed twice in LB medium supplemented with 500 µM 2,2-dipyridyl. Washed bacteria were then inoculated to a final optical density (OD) of about 0.01 into LB medium containing 2,2′-dipyridyl (450 µM) only or supplemented separately with hemoglobin (2 µM), hemin (16.5 µM), ferritin (0.3 µM), transferrin (1.5 µM) or lactoferrin (1.5 µM). Knowing that both the content of iron per molecule and the biochemical structure largely vary among these sources; the concentration of these iron sources were determined to give the optimal conditions for *B. cereus* growth (data not shown). Cultures were grown at 37°C with aeration and bacterial growth was monitored at an optical density of 600 nm over 10 hours.

### Growth index

*B. cereus* wild-type strain, the mutant strain *B. cereus* Δ*ilsA* and the complemented strain *B. cereus* Δ*ilsA* pHT304Ω*ilsA* were inoculated into LB medium supplemented with 200 µM 2,2′-dipyridyl and grown overnight. Overnight cultures were inoculated into LB medium supplemented with 200 µM 2,2′-dipyridyl at a final OD of 0.1. After incubation for 8 hours at 37°C, the resulting cultures were washed with 500 µM 2,2′-dipyridyl and inoculated at an OD of about 0.01 into fresh iron-restricted LB medium containing the appropriate antibiotics in the presence of 450 µM 2,2′-dipyridyl with or without either hemoglobin (2 µM), or hemin (16.5 µM), or ferritin (0.3 µM) or FeCl_3_ (200 µM) as a positive control. After 24 h of incubation, bacterial growth was recorded by measuring the absorbance at 600 nm (OD_600 nm_) and the data were generated as the growth index (growth in iron-restricted LB with or without the iron sources divided by growth in iron-rich LB). The assays were repeated three times.

### Enzyme Linked Immuno Sorbent Assay (ELISA)

The binding assay was performed by using increasing amounts of hemoglobin (0–180 nM) and ferritin (0–180 nM) for coating 24-well ELISA plates with buffer (0.1 M phosphate buffer pH 7.4). After incubation for 18 hours at 4°C, wells were blocked with 1% BSA dissolved in the coating buffer for 1 h at 37°C. After blocking and between each further step, wells were washed three times with 0.15 M NaCl dissolved in the coating buffer with 0.1% Tween 20. Purified IlsA was added at a concentration of 30 nM and incubated with hemoglobin and ferritin for 2 hours at 37°C. Interaction between hemoglobin and IlsA or ferritin and IlsA was detected with the polyclonal anti-IlsA antibody at a 1∶500 dilution and the horseradish peroxidase-labelled secondary anti-rabbit immunoglobulin G (IgG) at a dilution of 1∶10 000. Binding of IlsA was quantified by measuring the conversion of the chromogenic substrate, otho-phenylendiamine dihydrochloride (OPD), to the coloured product based on optical density readings at 492 nm. Readings were transformed and used for estimation of the dissociation constant, *K*_d_, using the computer program Origin.

### Surface Plasmon Resonance (SPR)

Real time binding kinetics experiments were conducted on a BIAcore 3000 (GE Healthcare Europe). GST-IlsA fusion protein was immobilized via its GST-tag in a flow cell of a CM5 sensor chip. For this purpose, a goat anti-GST antibody was first immobilized on the sensor chip using amine-coupling chemistry. The surface was activated for 7 min with a mixture of 0.05 M NHS and 0.2 M EDC. Anti-GST was then covalently linked to the surface giving up to 8000 resonance units (RU). Ethanolamine (1 M, pH 8.5) was injected for 7 min to block the remaining activated groups. Capture of GST-IlsA (1 µM) was performed at an injection flow of 5 µl min^−1^ in 0.1 M Phosphate buffer (pH 7.4), until stabilization of the immobilization level (6–8 min). For binding kinetics, a concentration of 200 nM of each IlsA ligand (hemoglobin, hemin, ferritin, and transferrin) was injected on the captured IlsA for 8 min and dissociation was registered for 10 min after the end of injections. Regeneration of the anti-GST capture antibody was achieved by a 2 min injection of glycine buffer (pH 2.2). The sensorgram from a cell with immobilized GST-IlsA was corrected by subtracting the response sensorgram from a cell with immobilized only anti-GST (reference surface) and normalized to a baseline of 0 RU. All measurements were performed at 20°C. Sensorgrams were analyzed using BIAevaluation Software. SPR tests were run 5 times on three different chips.

### Heme detection by chemiluminescence

Hemin was dissolved immediately before use in a minimal volume of 0.1 M NaOH and diluted with phosphate buffer (Na_2_HPO_4_ 0.1 M, pH 7.4) to a concentration of 10^−4^ M. Twenty microliters of purified GST-IlsA (2.5×10^−7^ M) were incubated with heme or with the buffer at room temperature for 30 min. Purified GST (2.5×10^−7^ M) was also used as negative control. Mixtures were separated by non-denaturing 8% PAGE in the absence of SDS at 4°C and the proteins were electrotransferred to a nitrocellulose membrane. The presence of heme complexed to IlsA was detected by chemiluminescence due to the heme peroxidase activity [36], using the Pierce ECL Super signal system.

### *In vivo* expression using the *gfp* reporter gene

In order to follow the expression of *ilsA in vivo*, we constructed a plasmid carrying the p*ilsA*-*gfp* transcriptional fusion. The plasmid pHT315-*gfp* was obtained by cloning the gene *gfp-mut1* into the plasmid pHT315 [35] between *Xba*I and *Hin*dIII restriction sites. *gfp-mut1* was amplified from the template plasmid pNF8 of *Listeria monocytogenes* [37] by using the primers F-gfp (5′-GCTCTAGAGAAAGGAGGTTATTAAAATGAGTAAAGGAGAAGAACTT-3′) and R-gfp (5′-CCCAAGCTTTTATTTGTATAGTTCATCCATGCCA-3′). The *ilsA* promoter region (p*ilsA*) was generated by PCR with oligonucleotide pairs Fw-p*ilsagfp* (5′-CCGGAATTCGGGCTTTTTTATTTTGTACC-3′) and Rv-I1 (5′-CCCTGAACAGTGTTCTCGG-3′) using the pHT304-*pivi29′-*I from the IVET screen [32] as a template. The PCR fragments were digested by *Eco*RI and *Xba*I and were inserted between the *Eco*RI and *Xba*I restriction sites of pHT315-*gfp* plasmid to give the plasmid pHT315-p*ilsA'-gfp*. The resulting plasmid pHT315-p*ilsA'-gfp* was cloned in *E. coli* and was then used to transform *B. cereus*. The strain harboring the plasmid pHT315-*pilsA*'-*gfp* was used to infect the lepidopteran larvae of *G. mellonella* orally in order to follow the kinetics of *ilsA* expression *in vivo*. The insect larvae were infected with 5×10^6^ mid-log phase bacteria (OD_600_ ∼1) in association with 3 µg of Cry1C toxin and incubated at 37°C, according to the protocol previously described [38]. Larvae were dissected and bacteria were recovered from the insect gut at 9 hours after infection and from the hemocoel at 24 hours after infection. The bacteria were then subjected to bright field (DIC) and epifluoresence microscope observation (NIKON Eclipse E600 equipped with UV mercury disposal and observed with an FITC filter). For a positive control we used the *B. cereus* strain harbouring the plasmid pHT315-p*aphA3'-gfp* that contains a transcriptional fusion between the constitutive promoter p*aphA3* obtained by PCR amplified from the pGDG783 plasmid [39] and the *gfp* gene (C. Nielsen-LeRoux, unpublished data).

### Growth kinetics of *B. cereus* in the hemocoel of *G. mellonella*

The growth (number of bacteria) was assessed at 2, 6 and 24 hours after injecting doses of 500 mid-log phase bacteria of the wild type or the *ilsA* mutant strains into the hemocoel of the larvae *G. mellonella*. At each time-point, three alive larvae were recovered, surface-sterilized by rinsing briefly in 70% ethanol, crushed, and homogenized separately in 10 ml of phosphate buffered saline (PBS) pH 7.4, using a polytron homogenizer (Kinematica, Switzerland). Serial dilutions were plated on LB medium, in order to estimate the number of bacteria present in the larvae. At each time point, the results are the means of nine independent larvae from three independent tests. The resulting data were analysed using the Student T-test.

### Virulence assays

Contribution of IlsA to *B. cereus* virulence was assayed by comparing the killing effect of the wild-type and the *ilsA* mutant *B. cereus* strains, after injecting them separately into the hemocoel of *G. mellonella* larvae. Last-instar larvae weighing about 200 mg were injected with 10 µl of mid-log phase bacteria suspended in PBS, using the microinjector (Buckard Scientific, UK.) as previously described [40]. Various doses of *B. cereus* wild-type and the *ilsA* mutant (10^3^ to 10^2^ bacteria/larvae) were used, and each dose was repeated three times on at least 20 larvae. A control group of larvae was injected with PBS only. Infected larvae were kept at 37°C and mortality was recorded after 24 hours. The LD_50s_ values were estimated using Probit analysis [41],[42]. This program tests for the linearity of dose-mortality curves, provides lethal doses and the slope of each dose-mortality line. It tests the parallelism of two or more dose-mortality lines and determines the virulence ratio between the bacterial strains. The ratio is considered to be significantly different from 1 (P<0.05) when the confidence limits do not include the value 1.

## Results

### Localization of IlsA on the bacterial surface

The analysis of the IlsA sequence indicated a possible SLH-domain (S-Layer Homology) presumably binding the protein to the peptidoglycan (Figure 1A) and our previous results showed that IlsA was expressed in iron depleted conditions [32], thus we analyzed its localization in such conditions. The localization of IlsA was determined by immunofluorescence using the polyclonal antibody anti-IlsA (Figure 1B). IlsA was detected on the surface of *B. cereus* wild-type grown in iron depleted condition (LB supplemented with iron chelator) (Figure 1B iv–vi), whereas IlsA was not present on the surface of either the wild-type strain grown in LB medium (standard conditions) (Figure 1B i–iii) or the mutant strain *B. cereus* Δ*ilsA* grown in iron depleted medium (Figure 1B vii–ix). Transformation of the *B. cereus* Δ*ilsA* mutant with the plasmid pHT304-*ilsA* restored the production of IlsA at the surface of the bacteria grown in iron-depleted medium (Figure 1B x–xii). In addition, we observed an accumulation of IlsA on the division site of the bacteria. These results indicate that IlsA is present on the surface of the bacteria and only in iron depleted conditions, which is in agreement with the iron regulated expression of *ilsA* [32].

### *B. cereus* uses hemoglobin, hemin and ferritin as iron sources

**Figure 2:**
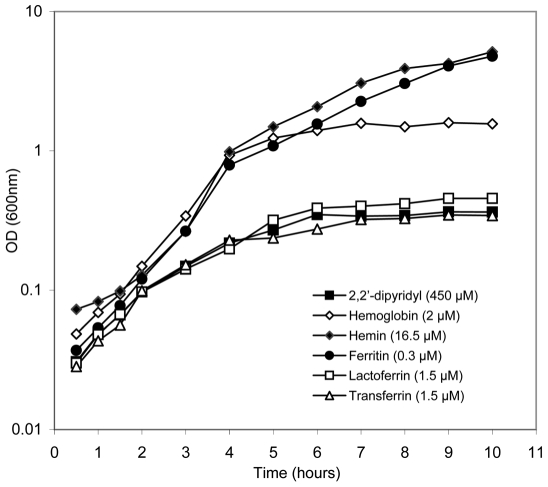
Growth kinetics of *B. cereus* wild-type using iron-rich host proteins as sole iron sources. *B. cereus* was grown in LB medium treated with 2,2′-dipyridyl without addition of iron sources (black square) or supplemented with hemoglobin (white diamond), or hemin (black diamond), or ferritin (black circle) or transferrin (white triangle) or lactoferrin (white square). Bacterial growth was determined by measuring the optical density (OD) at 600 nm. Curves are representative of three independent experiments.

The ability of *B. cereus* ATCC 14579 to utilize host iron sources has not been previously investigated. To determine which iron sources *B. cereus* is able to use, we measured its ability to grow on hemoglobin, hemin, ferritin, transferrin and lactoferrin, in iron depleted LB medium (Figure 2). *B. cereus* growth was very weak in iron depleted condition, but the addition of either hemoglobin, or hemin, or ferritin to the iron depleted medium increased significantly the growth kinetics of *B. cereus*. In contrast, transferrin and lactoferrin addition to iron depleted medium yielded only growth of *B. cereus* similar to that in iron depleted medium.

This result indicates that *B. cereus* is able to use hemoglobin, hemin and ferritin as iron sources for growth, whereas transferrin and lactoferrin cannot be used.

### IlsA is required for efficient iron acquisition from hemoglobin, hemin and ferritin

**Figure 3:**
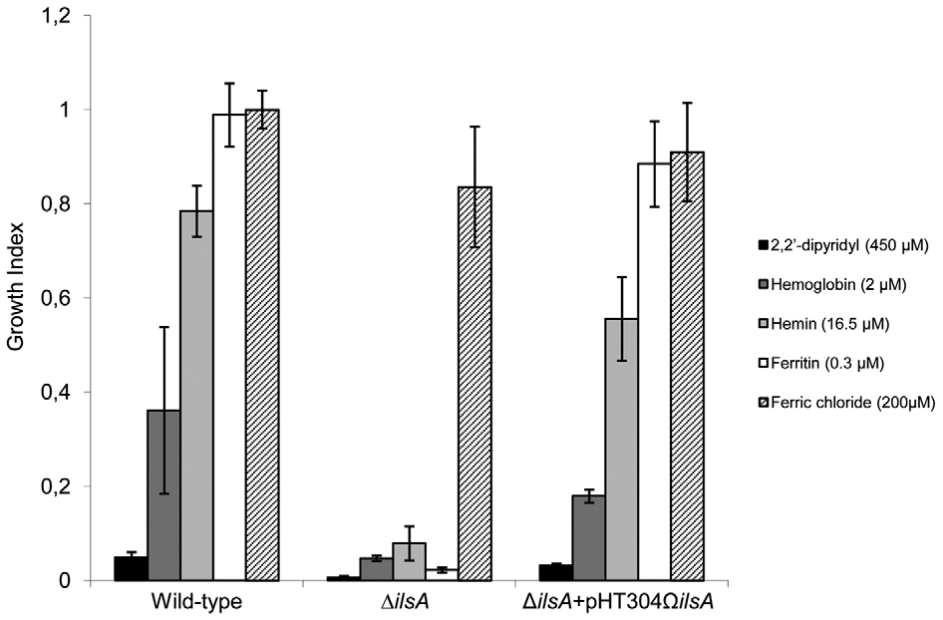
Growth index phenotype of *B. cereus* wild-type strain and the mutant *B. cereus* Δ*ilsA* using iron-rich host proteins as the sole iron sources. *B. cereus* wild-type, the mutant Δ*ilsA* and the complemented Δ*ilsA*+pHT304Ω*ilsA* strains were grown in iron restricted medium treated with 2,2′-dipyridyl (450 µM) or supplemented with host iron sources: hemoglobin (2 µM) or hemin (16.5 µM) or ferritin (0.3 µM) for 24 h. Ferric chloride (200 µM) was used as a control for inorganic iron source. The optical density was recorded at 600 nm and the growth index was quantified (growth in indicated conditions, compared to growth in LB (iron-rich medium). The mutant Δ*ilsA* strain was unable to grow in the presence of any of the tested host iron sources and complementation of *ilsA* to the mutant strain Δ*ilsA* restores the bacterial growth phenotype. The mutant Δ*ilsA* strain was able to grow with ferric chloride. Results are the means of three independent experiments and error bars indicate the standard deviations of the mean.

To test the hypothesis that IlsA is important for iron scavenging from the host iron sources used by *B. cereus*, the growth rates of the wild-type and *ilsA* mutant strains were compared after inoculation in iron depleted medium where either hemoglobin, hemin or ferritin were provided as the sole iron source. Bacterial growth was measured by spectrophotometric analysis of culture samples at an optical density of 600 nm (OD_600_) (Figure 3). Iron depleted LB did not support the growth of the wild-type and the Δ*ilsA* mutant strains, confirming that iron is an essential nutrient for *B. cereus* growth. In contrast, the wild-type strain grew well in iron depleted LB that had been supplemented with hemoglobin, hemin or ferritin, while *B. cereus* lacking *ilsA* showed significant growth defects in these media. However, the mutant strain grew as the wild-type when iron was provided in its inorganic form (FeCl_3_). Therefore, IlsA is not required for the uptake of inorganic iron. To verify that this phenotype was specifically due to *ilsA*, the mutant strain was complemented with a plasmid harboring *ilsA*. The growth of the bacteria was restored to the wild-type level when hemoglobin, hemin or ferritin was added to iron depleted LB. These data indicate that IlsA is very important for efficient growth of *B. cereus* under iron-restricted conditions; they suggest that IlsA is involved in iron acquisition from the host during infection.

### IlsA binds hemoglobin and ferritin

**Figure 4:**
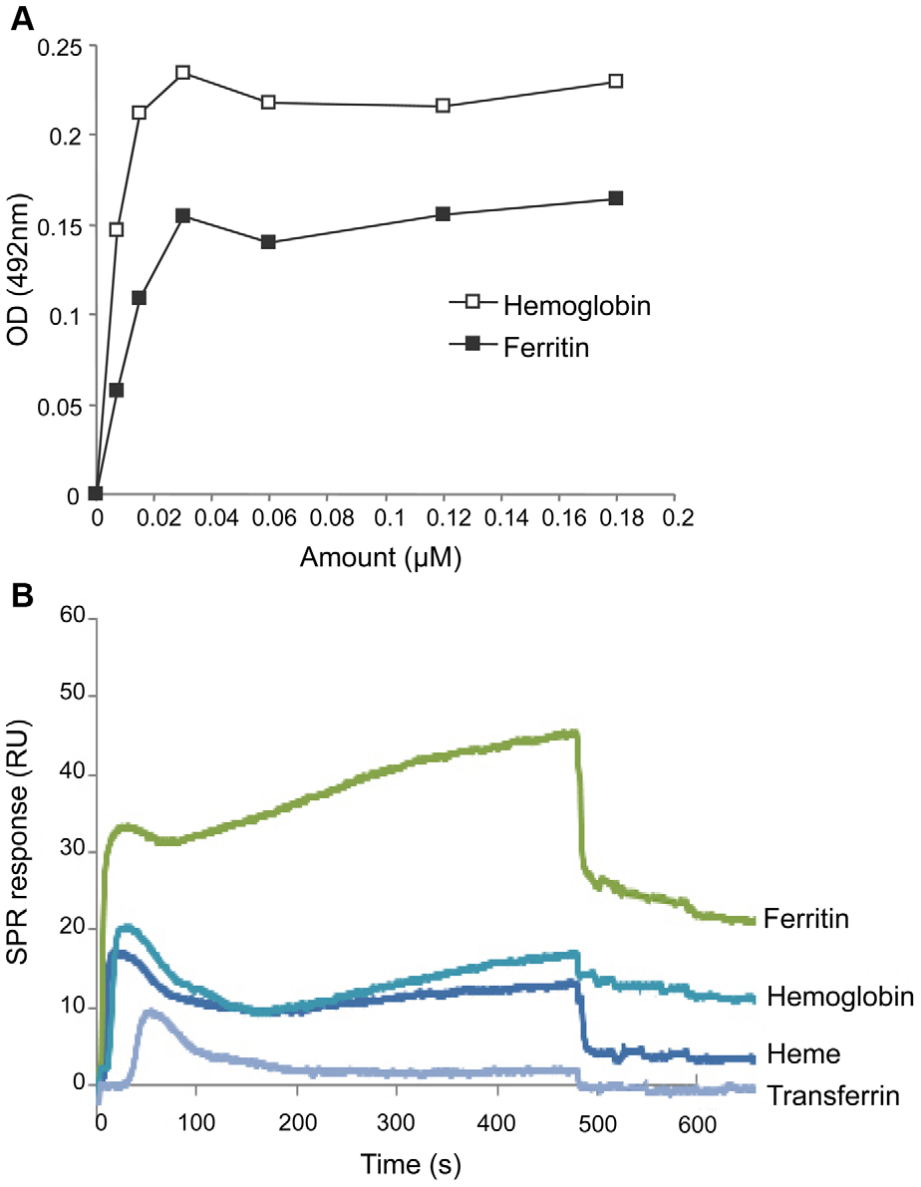
IlsA binds hemoglobin and ferritin. (A) Binding of purified GST-IlsA to hemoglobin and ferritin was performed in an ELISA based assay. Hemoglobin (white square) and ferritin (black square) were coated on ELISA plates at increasing amounts, and IlsA was added at 3 pmole. Binding was quantified by measuring the absorbance after incubation with anti-IlsA and the secondary antibody peroxidase labelled. Data shown are the mean of three independent experiments. (B) Real-time kinetics of interaction between heme, ferritin, hemoglobin and transferrin with GST-IlsA fusion protein. Overlay plot of sensorgrams corresponding to interaction of these various proteins with GST-IlsA fusion protein. GST-IlsA fusion protein was first captured to saturation by anti-GST antibody linked covalently to the carboxymethyldextran chip. Then various iron sources (200 nM) were injected for 8 min after which dissociation was followed. Note the absence of transferrin binding. The sensorgrams are representative for five experiments.

To address the question of whether IlsA can directly interact with hemoglobin and ferritin, we performed binding studies using the purified recombinant protein GST-IlsA. In these assays, increasing amounts of hemoglobin and ferritin were immobilized on ELISA plates (hemin can not bind to these plates) and IlsA was added. The amount of bound IlsA was detected using anti-IlsA polyclonal antibody. By comparing the signal intensities generated by the binding of IlsA to the different proteins, we detected a slightly stronger binding of IlsA to hemoglobin (*K*_d_ = 3±1 nM) relative to ferritin (*K*_d_ = 10±3 nM) (Figure 4A). However, the Student T-test showed no significant difference (p<0.05) for IlsA to bind hemoglobin and ferritin.

To investigate whether binding also occurs in a real time interaction, we used the Surface Plasmon Resonance (SPR) system. The interaction between chip immobilized GST-IlsA and different host iron-rich molecules (hemin, hemoglobin, ferritin and transferrin) under flow conditions shows typical SPR curves (Figure 4B). These SPR curves are from one experiment, but the assays were run 5 times on 3 different chips for which the curves shape were similar to the ones shown. Computational analysis showed that our data don′t fit into a typical association-dissociation model with a 1∶1 molar ratio. This is probably resulting from protein multivalencies and potential steric hindrance of the various sizes of the ligands (ferritin 440 KDa, hemoglobin 68 KDa and heme 651 Da). Then, quantitative information about the relative stability of the complex, was deduced from the dissociation phase only, independent from the analyte concentration. Then, assuming a simplified kinetic scheme, the indicative kinetic dissociation constants (k_off_, expressed in s^−1^) were calculated: 1.71±0.83×10^−3^ for ferritin, 1.48±0.98×10^−3^ for hemoglobin and 1.34±0.44×10^−3^ for hemin. Thus, the results showed similar k_off_ values for the three iron sources. These findings are in accordance with the ELISA experiments showing that soluble ferritin and hemoglobin bound to immobilized IlsA, but transferrin did not (result not shown). The lack of direct interaction between IlsA and transferrin could be expected, because the growth experiment (Figure 2) indicated that this protein was not used by *B. cereus* as an iron source.

### IlsA binds heme

**Figure 5:**
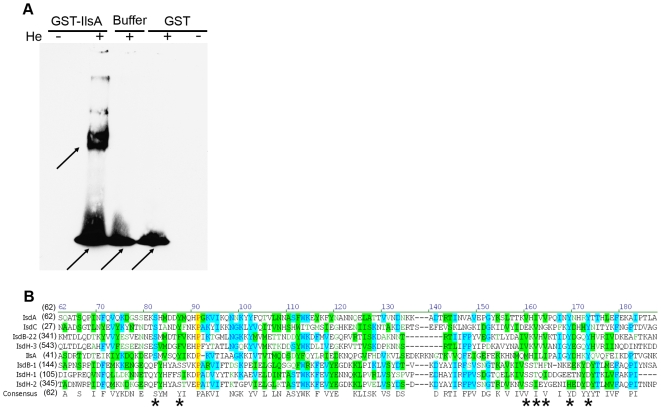
Hemin binding analysis of purified IlsA. (A) Chemoluminescent detection showing binding of GST-IlsA to hemin (He). Purified GST-IlsA or GST incubated either with hemin (+) or without (−) as indicated on the top of the lane, were migrated on non-denaturing PAGE and transferred to a nitrocellulose membrane. Phosphate buffer alone was also incubated with hemin (+) as a control. After reaction with an ECL reagent system, proteins with hemin-binding properties are detected on chemoluminescent-sensitive film. Upper arrow corresponds to GST-IlsA binding to hemin and lower arrows to hemin alone. (B) Multiple sequence alignments of NEAT domains of IlsA from *B. cereus* and Isd proteins from *S. aureus*. Positions shaded in yellow are identical in 100% of the aligned sequences, blue and green are identical or similar, respectively, in at least 50% of the aligned sequences. The first four letters of the sequence represent the protein identity. Asterisks represent the amino acids required for heme binding. Numbers 1 to 3 represent the NEAT domain numbered from the N-terminus out of the total number of NEAT domains in that protein. The accession numbers of Isd proteins from *S. aureus* are as follows: IsdH (Q99TD3), IsdB (Q7A656), IsdA (Q7A655), IsdC (Q7A654). The accession number of IlsA from *B. cereus* ATCC 14579 is NP_831113. NEAT domains were identified in the Pfam database. Alignments were generated in vector NTI advance 10 (Invitrogen), using a BLOSUM matrix with gap opening and extension penalties of 15 and 2, respectively.

To determine whether IlsA in solution binds to heme or another component of hemoglobin, we incubated purified GST-IlsA and GST (negative control) with hemin (10^−4^ M) and analyzed the binding reaction on a non-denaturing polyacrylamide gel system (Figure 5A). This allows the separation of free heme, purified proteins and heme-loaded proteins without dissociating heme from the proteins. Proteins were transferred to a nitrocellulose filter, and heme peroxidase activity was detected by chemiluminescence. This method is the most sensitive test available for detecting heme bound to proteins [36]. Our results showed that GST-IlsA could bind heme-iron, whereas GST alone cannot. This is in agreement with the result of the multiple sequence alignment of the NEAT domain of IlsA with those found in Isd proteins of *S. aureus* (Figure 5B). Indeed, this comparison indicates that the three conserved residues (Ser^82^, Tyr^166^ and Tyr^170^) present in IsdA, which are essential for heme interaction [11],[12], are also present in IlsA. Furthermore, the residues Val^157^, Ile^159^ and Val^161^ that form the hydrophobic heme-pocket in IsdA are either conserved in IlsA or replaced with similar hydrophobic amino acids. The conservation in IlsA NEAT domain of amino acid residues known to interact with heme, suggests that IlsA binds heme through the NEAT domain.

### *ilsA* is strongly expressed in the hemocoel of infected larvae

**Figure 6:**
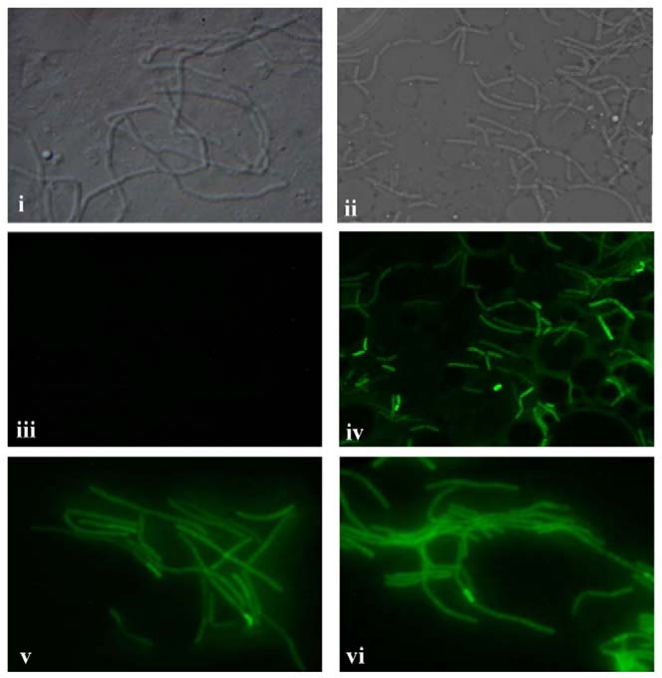
Analysis of *ilsA* expression *in vivo*. Microscopic observations by Bright-field DIC (panels i,ii) and epifluorescence (panels iii–vi) were performed on *B. cereus* carrying pHT315-p*ilsA*'*gfp* isolated at 9 hours after infection from the intestine of alive larvae (panels i,iii) and at 24 hours after infection from the hemocoel of dead larvae (panels ii,iv). Green bacteria indicate expression of *gfp* due to the activation of the *ilsA* promoter. *B. cereus* carrying pHT315-p*apha3*'*gfp* was used as a control for its constitutive expression of GFP (panels v,vi).

The IVET screen showed that *ilsA* was strongly expressed during infection of the insect larvae [32]. However, it was not determined at which stage of the infection and in which tissue *ilsA* expression occurred. To determine where and when *ilsA* is expressed *in vivo*, larvae were infected with *B. cereus* harbouring a plasmid that contained a transcriptional fusion of the promoter p*ilsA* and the *gfp* reporter gene. Bacteria recovered from the larvae at various times post-infection were examined under brightfield (Figure 6i–ii) and epifluoresence microscopy (Figure 6iii–vi). No fluorescence was observed in bacteria isolated from the intestine of living larvae after nine hours of infection (FIGURE 6iii). In sharp contrast, *B. cereus* bacteria isolated from the hemocoel of dying larvae after 24 hours of infection, showed strong fluorescence (FIGURE 6iv), thus indicating that *ilsA* is highly expressed in the hemocoel. Indeed, iron is not accessible to bacteria in the hemocoel due to the presence of proteins that bind iron such as transferrin and ferritin [43],[44]. *B. cereus* containing the plasmid (pHT315-p*aphA3'-gfp*) with the constitutively expressed *gfp* gene, showed a strong expression in both the intestine and hemocoel of the infected larvae (Figure 6v–vi).

### IlsA is important for bacterial growth and adaptation of *B. cereus* in the hemocoel

**Figure 7.-:**
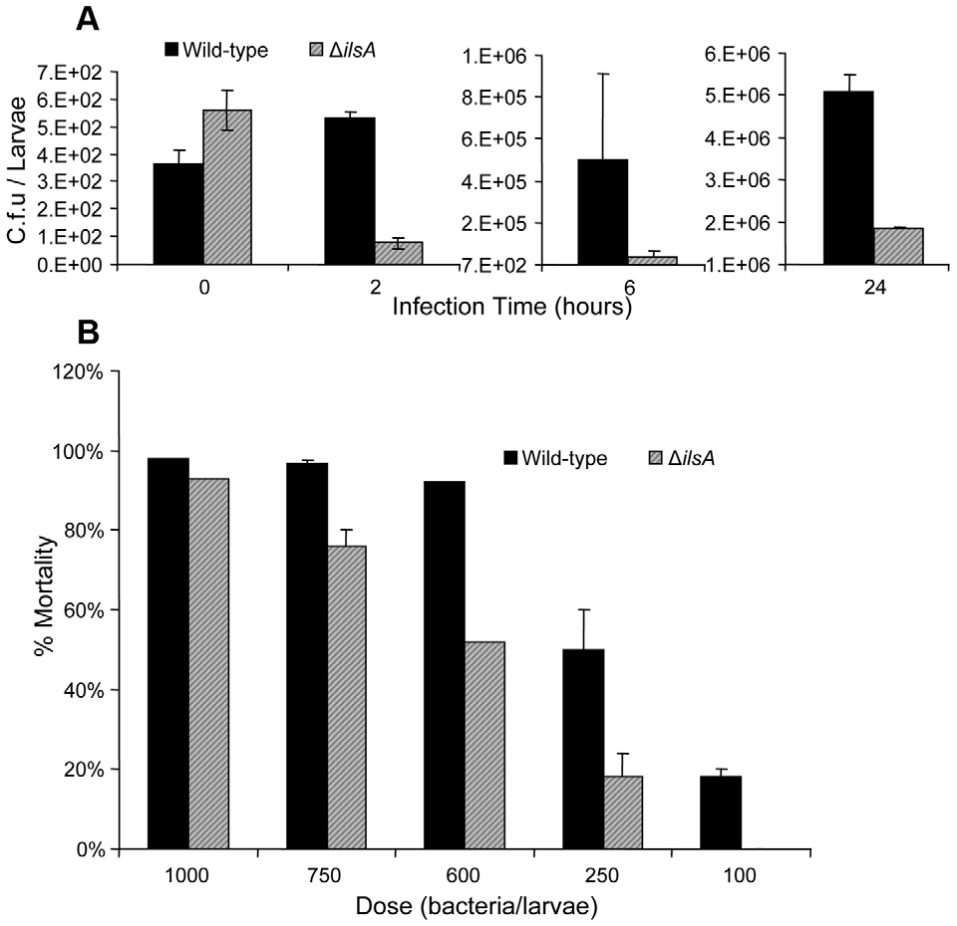
Ils*A* is required for *B. cereus* infection in the model *G. mellonella*. (A) Effect of *ilsA* mutation on the growth of *B. cereus* after intrahemocoelic injection in *G. mellonella*. Wild-type and the mutant Δ*ilsA* strains were injected separately into the hemocoel of last-instar larvae at a dose of 5×10^2^ mid-log phase bacteria. Bacteria were counted at various times after injection (2, 6 or 24 h) from living larvae. Time 0 h is the real dose of each strain injected in the hemocoel. Results are the mean of bacterial counts from nine different larvae and error bars indicate standard errors of the means. (B) Effect of *ilsA* mutation on the virulence after intrahemocoelic injection in *G. mellonella*. Last-instar larvae were injected with various doses (10^2^ to 10^3^) of mid-log phase bacteria. Mortality was evaluated after 24 h of injection of *B. cereus* wild-type (bold histogram) and *B. cereus* Δ*ilsA* (grey histogram). Results are mean values of four independent experiments and error bars indicate the standard errors of the means.

Growth of the wild-type and Δ*ilsA* mutant strains was assessed by homogenization of whole larvae at several time points after injection into the hemocoel of *G. mellonella* (Figure 7A). A dose of ∼500 mid-log phase bacteria (estimated as colonies forming units (cfu)) was injected and used as the start point. After 2 hours of injection, the number of the Δ*ilsA* bacteria in the insect larvae decreased drastically (75.5 cfu), while the number of the wild-type bacteria remains stable (530 cfu). After 6 hours, *B. cereus* Δ*ilsA* had a growth of about 10-fold less (39.4×10^3^ cfu) than the *B. cereus* wild-type strain (50.3×10^4^ cfu). After 24 hours, the growth of the mutant strain (18.5×10^5^ cfu) was about 2,5-fold less than the *B. cereus* wild-type strain (50.7×10^5^ cfu). The growth of *B. cereus* Δ*ilsA* was significantly different (p<0.05) from the *B. cereus* wild-type strain at 2 hours and 24 hours after injection.

To test if the growth defect of the mutant strain affects the virulence of *B. cereus* in the hemocoel, we analyzed virulence tests where bacteria were directly injected into the hemocoel of *G. mellonella*. After 24 hours of infection with various doses of mid-log phase bacteria (10^2^ to 10^3^), the number of dead larvae was recorded (Figure 7B). The 50% lethal doses (LD_50s_) of the wild-type and of the Δ*ilsA* mutant strains were assessed by Probit analysis and are shown as histograms. The wild-type strain had an LD_50_ of 2.2×10^2^ (95% confidence limits between 1.8×10^2^ and 2.6×10^2^) bacteria per larva and the mutant showed an LD_50_ value of 4.8×10^2^ (95% confidence limits between 4.1×10^2^ and 5.7×10^2^) bacteria per larva. A decrease of 2.2-fold (range 1.59 to 3.07 at the 5% confidence level) was determined by comparing the two dose-response regression lines. However, an additional statistical analysis (Student T-test) performed on each dose, indicates only a significant difference (p<0.05) at the lowest dose (100 bacteria/larvae). Then, the importance of IlsA by this route of infection seems less than by the oral route, as earlier reported [32], although IlsA helps *B. cereus* to survive in the hemocoel, as shown in the above *in vivo* growth analysis. These results indicate that IlsA is important for the growth and the survival of *B. cereus* in the hemocoel following injection or natural injury.

## Discussion

The ability of living organisms to sequester free iron is not only necessary for protection against iron toxicity but it is also an innate resistance mechanism of hosts (mammals or insects) to fight infections [45],[46]. Iron incorporated into heme is the most abundant iron source in the mammalian host, being mainly associated with the oxygen-carrying molecule, hemoglobin, sequestered within erythrocytes. Ferritin, the major iron storage protein is a large molecule, composed of several subunits, containing about 4500 ferric ions per molecule, is present not only in mammals but also in almost all living organisms including insects, plants (phytoferritin) and bacteria (bacterioferritin) [47]. In insects, notably in *G. mellonella*, ferritin is present in high amounts in hemolymph and in hemocytes and may play a role in iron transport in addition to iron storage [44],[48]. Thus, free iron is not available and bacteria are iron restricted whatever their hosts. Therefore, they must be able to chelate and actively take up any available iron in order to grow and successfully infect their hosts. It has been shown that expression of the majority of genes involved in iron acquisition and metabolism, such as those coding for surface receptors, transport proteins, some hemolysins and cytotoxins, are controlled by the repressor Fur [1],[3]. In this study, we show that *B. cereus* can use hemoglobin, hemin and ferritin as iron sources through the surface receptor IlsA that also belongs to the Fur regulon. We show that *B. cereus* strain ATCC 14579 is not able to use lactoferrin or transferrin for its growth, which is in accordance with Sato *et al.* 1999 [30],[31] and in disagreement with Park *et al*. 2005 [29]. This result suggests that there might be strain variations, related to iron acquisition, but also that this *B. cereus* strain might be unable to cope with the antibacterial activity of lactoferrin [49], since higher concentrations of lactoferrin affected its growth. Hemoglobin and ferritin are intracellular iron rich proteins and must be released from cells in order to be used by *B. cereus* for its growth. It has been shown that *B. cereus* produces a large variety of cytotoxic proteins (Hbl, Nhe, CytK, Clo and HlyII) able to lyse various eukaryotic cells including erythrocytes [50]–[52]. Expression of these cytotoxic proteins with the exception of hemolysin II (HlyII), is regulated by the pleiotropic regulator PlcR [24],[53]. Then, all these factors may contribute to release hemoglobin, heme and ferritin from cells following cell lysis. These iron-binding molecules can then be used for *B. cereus* growth after direct binding to the surface receptor, IlsA. Our results show that purified recombinant IlsA can bind hemoglobin and ferritin *in vitro*. In addition, IlsA also binds heme alone, suggesting that the interaction between IlsA and hemoglobin is due to its interaction with heme. How IlsA binds heme is currently unknown; however, studies from other NEAT proteins have identified conserved tyrosine residues and hydrophobic residues that form a heme pocket, which mediates heme binding [11],[12]. These residues are also present in the IlsA NEAT domain, suggesting that IlsA mediates heme binding through this NEAT. The interaction with ferritin is much less evident, although it has previously been reported that the NEAT protein IsdA in *S. aureus* binds to several non-heme host proteins, including the ferric iron carrier transferrin [54], and several matrix and plasma proteins [55]. IlsA is a more complex protein than IsdA because of the LRRs domains. These domains are found in a large range of proteins with different functions and are known to bind structurally unrelated protein ligands [56],[57]. In Gram positive bacteria, only few LRR-containing proteins have been characterized to date, especially in *Listeria monocytogenes*, *Streptococcus pyogenes* and *Enterococcus faecalis*, where they are involved in the interaction with the host [57]. Thus, it is tempting to speculate that the LRR domains of IlsA are involved in the binding to ferritin. To our knowledge, IlsA is the first LRRs protein that has a function in iron uptake. A recent study revealed that a surface protein of *Candida albicans*, Als3, is essential for iron acquisition from ferritin by binding to the fungal hypha [58]. However, there is, no obvious structural homology, between Als3 (family of agglutinin like adhesion molecules) and IlsA. Moreover, the authors did not report any direct *in vitro* binding between ferritin and Als3. Thus, in depth structural studies related to the interaction between IlsA and ferritin are needed to elucidate this aspect.

Our results suggest that IlsA is a key protein of a new iron/heme acquisition system in *B. cereus*. To achieve the transport of ferric iron from ferritin and heme across the cell wall and the cytoplasmic membrane into the cytoplasm, IlsA has to interact with other bacterial partners. The iron-regulated surface determinants (Isd) involved in heme acquisition and transport has been studied in several Gram-positive bacteria especially in *S. aureus* and *B. anthracis*. In *S. aureus*, the Isd system is composed from cell wall anchored proteins acting as hemoprotein receptors that are in vicinity of an iron transporter ABC systems [9],[10]. Unlike *S. aureus*, the heme uptake system Isd in *B. anthracis* depends on secreted NEAT domains proteins in the extracellular environment and also on those located in the bacterial envelope which enable heme transfer to an ABC transporter system and then into the cytoplasm [13],[14,13].

**Figure 8:**
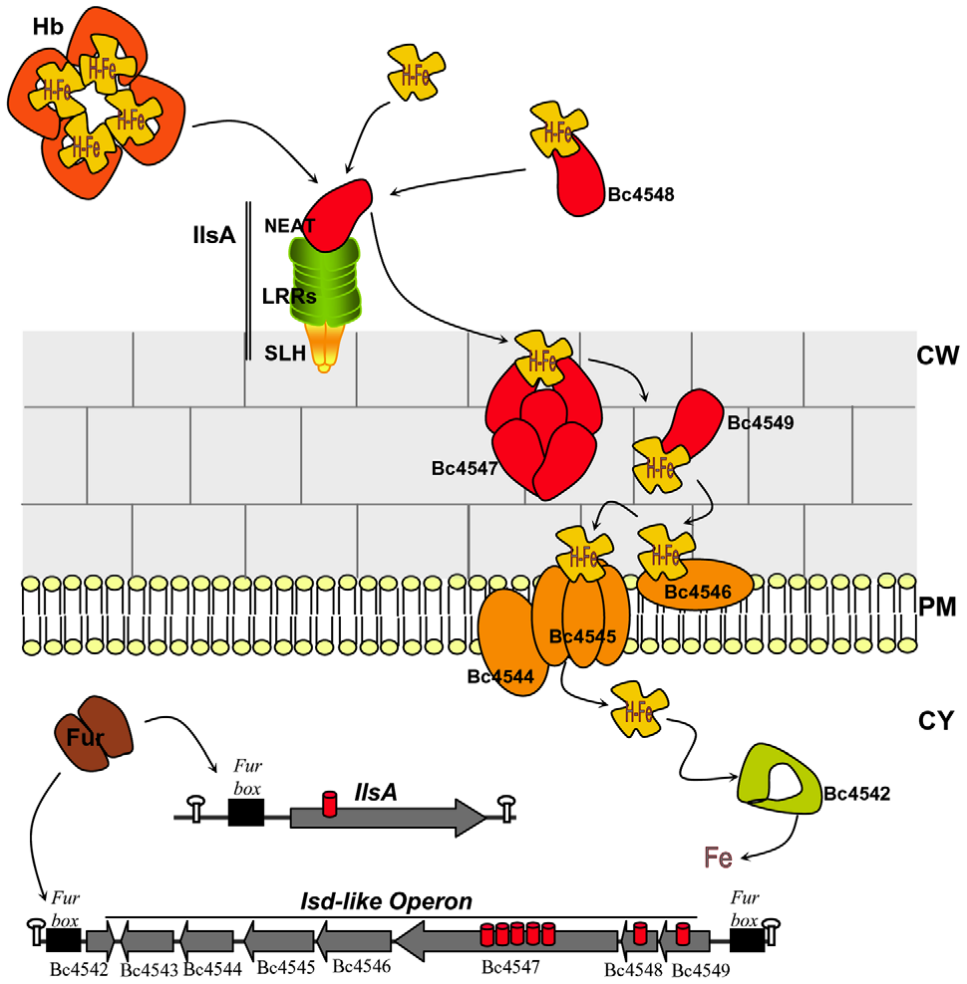
Model for heme iron acquisition in *B. cereus*. In the host, *B. cereus* is in iron restrictive environment and the global iron regulator Fur is removed from its binding site upstream of *ilsA* and *isd*-like genes, leading to their expression. IlsA and Isd-like proteins are localized in their appropriate cellular locations. IlsA binds heme (H-Fe) directly from hemoglobin (Hb) or indirectly through Bc4548. Heme will then be transferred to Bc4547, Bc4549 and finally to the ABC transport system (Bc4544-45-46) located in the plasma membrane. Once heme is present in the cytoplasm, the monooxygenase (Bc4542) catalyzes the degradation of the heme and the released iron can be used for *B. cereus* growth. Arrows indicate the orientation and approximate sizes of the open reading frames. Potential transcription terminators are represented. The putative *fur boxes* in the promoter region upstream of *ilsA* (BC1331) and the *Isd*-like operon are indicated. Each cylinder indicates a NEAT domain. CW  =  cell wall, PM  =  plasma membrane, CY  =  cytoplasm.

The heme scavenging strategy evolved by *B. cereus* seems to be different from those described in *S. aureus* and *B. anthracis*, since it involves the surface protein IlsA, which contains SLH domains that enable its binding to peptidoglycane [59]. Homologs of IlsA are found in *B. anthracis* (belonging to Bsl surface proteins), but none of them possess the three conserved domains (NEAT, LRR, SLH) at the same time like IlsA. In addition to three SLH domains, BslK presents a NEAT domain, while BslL contains LLR domains [60]. Then, since *B. anthracis* can use heme as an iron source via the Isd uptake system [61], it might suggest that IlsA homologs in *B. anthracis* do not function in the same way as in *B. cereus*. Thus, on the basis of our studies and those from other Gram-positive bacteria, we propose a model (Figure 8) where IlsA interacts with NEAT proteins belonging to the Isd system to ensure heme transport through the bacterial envelope into the cytoplasm. *In silico* analysis of the *B. cereus* ATCC 14579 genome reveal, in addition to IlsA, several genes (Bc4547, Bc4548, Bc4549) encoding NEAT domain proteins. The protein encoded by Bc4548 contains a signal peptide, but lacks obvious anchoring motif. This protein, which has 98% identity with IsdX1 of *B. anthracis* [13],[14], may function as a secreted hemophore that captures heme from hemoglobin. The proteins encoded by Bc4547 and Bc4549 with the anchoring motifs NSKTA and NPKTG, respectively, may be the substrates of the putative sortase B (Bc4543) and being located in variable positions throughout the peptidoglycan. These proteins have 87% identity with IsdX2 [14] and 98% identity with IsdC of *B. anthracis* [61] respectively. Similar to *S. aureus* IsdC [62],[63] and *B. anthracis* IsdC [61], we propose that Bc4549 occupies a critical position enabling the transfer of heme to the iron permease system (Bc4544, Bc4545, Bc4546), followed by transport across the bacterial membrane. Finally, a putative intracellular monooxygenase, encoded by Bc4542 degrades heme to liberate iron to be used by *B. cereus*. The presence of IlsA is crucial for this iron-uptake system, since its disruption abolishes the ability of *B. cereus* to grow despite the presence of hemoglobin, hemin and ferritin. The mechanism by which IlsA permits the uptake of iron from ferritin is certainly not the same as from heme. We speculate that IlsA might be able to destabilize the ferritin structure, via a possible interaction with the IlsA LRR domains. The structural modification may permit other factors, such as reductases [64] or proteases [65] to liberate iron (Fe^3+^) that can then be captured by siderophores, such as petrobactin or bacillibactin [26] and transferred by an iron uptake-system into the cytosol. Studies are ongoing to investigate this hypothesis.

To understand the contribution of IlsA in the pathogenesis of *B. cereus*, we analyzed its expression during infection using *gfp* transcriptional fusion. The results showed that *ilsA* is highly expressed in the hemocoel where free iron is not accessible to bacteria, being bound to proteins such as ferritin [44]. IlsA is also expressed in collected hemocoel and when bacteria are directly injected into the hemocoel (results not shown), indicating that the expression of *ilsA* is not depending on a dying insect. Then, expression of *ilsA* could be initiated in response to iron depleted conditions and/or due to bacterial sensing of ferritin, thus enabling capture of iron from this source and subsequent use by *B. cereus* for growth and survival within *G. mellonella*. Moreover, disruption of *ilsA* decreases the growth and the virulence of *B. cereus* after injection into the hemocoel. However, the effect of IlsA on the virulence of *B. cereus* was larger following infection by oral route [32], compared to what we found here, following injection into the hemocoel. This can be explained by a combination of several factors among which one might rely on the activation of the ferritin production. Assuming when the bacteria are entering the hemocoel from the intestinal lumen, the innate sensing (production of antimicrobial peptides, ferritin etc.) has been turned on for a while [66] and thus less free iron is available. In this situation, the *ilsA* mutant cannot grow as well as the wild-type. A second point is reliant to the dose dependent *in vivo* growth capacity of the *ilsA* mutant, reported here (Figure 7B). Indeed, unpublished histopathological studies (Nielsen-LeRoux *et al*.), have shown that bacteria, from the intestinal lumen, enter the hemocoel by small numbers which can be considered equivalent to a low dose of hemocoel injected bacteria.

Together, these observations indicate that IlsA is an important factor required for adaptation within an insect host especially during the early stages of infection, as soon as the bacteria enter the hemocoel and before iron ions are released from degraded tissues. Thus, IlsA is the first host iron receptor in *B. cereus* shown to contribute to *in vitro* growth by using hemoglobin, heme and ferritin as an iron source, as well as contributing to virulence *in vivo* . To our knowledge, it is also the first time that an extracellular pathogen has been shown to use ferritin for its growth in physiological conditions that are correlated with host infection. The intracellular bacteria *Neisseria meningitidis* has been reported to degrade ferritin by manipulating the cellular machinery and the lysosomal activity [67] and *L. monocytogenes* was reported to use ferritin *in vitro* via a reductase activity [64].

In this study, we provide evidence that IlsA is necessary and sufficient to mediate interaction and iron acquisition from several iron-binding proteins. The exceptional structure of IlsA (NEAT and LRRs) probably explains its ability to bind to these different ligands. Moreover, since IlsA-like proteins are restricted to *B. cereus* group bacteria, it may be a concrete example of how bacteria create molecules adapted to their particular surface structure. Indeed, the SLH domain of IlsA permits binding to the peptidoglycan via a mechanism often found in this bacterial group [68]. Further investigations are needed for a complete understanding of the mechanism of iron-uptake through IlsA. Detailed description of the mechanism by which IlsA allows *B. cereus* to acquire iron from hemoglobin, heme and ferritin will provide a better understanding not only of *B. cereus* pathogenesis but also for infections caused by other extracellular Gram-positive pathogenic bacteria.

In conclusion, our investigations show how a bacterial adaptation factor can be directly correlated with a certain level of virulence. Furthermore, they provide insights into the host adaptation and the evolutionary ecology of the *B. cereus* group bacteria, including the mammalian pathogen *B. anthracis* and the entomopathogen *B. thuringiensis*. Since identified homologs of IlsA are restricted to the *B. cereus* group, which is composed of closely related bacteria able to colonize diverse hosts, IlsA may be a key factor allowing colonization of these different environmental niches. *B. cereus* and *B. thuringiensis* can cause infection in insects, mice and other mammals [17],[25] while there is little evidence that *B. anthracis* can infect invertebrates [69]. Altogether, our results show the first steps of a new and unique mechanism of iron acquisition, which is specifically adapted for interaction with two different iron-rich host proteins found in vertebrates and invertebrates.

## Supporting Information

Figure S1 Purification of IlsA. Coomassie-stained 10% SDS-PAGE analysis of the purified GST-IlsA and GST proteins from recombinant *E. coli*. Lane M, molecular weight markers in KDa. Purified GST-IlsA and GST with apparent molecular weights shown on the gel, was loaded in lane 1 and 2 respectively.
(0.51 MB PDF)
