# Supplementary figures and images for: IlsA, A Unique Surface Protein of Bacillus cereus Required for Iron Acquisition from Heme, Hemoglobin and Ferritin
Source: PLoS Pathog. 2009 Nov 26;5(11):e1000675. doi: 10.1371/journal.ppat.1000675 (PMC2777315; doi:10.1371/journal.ppat.1000675)

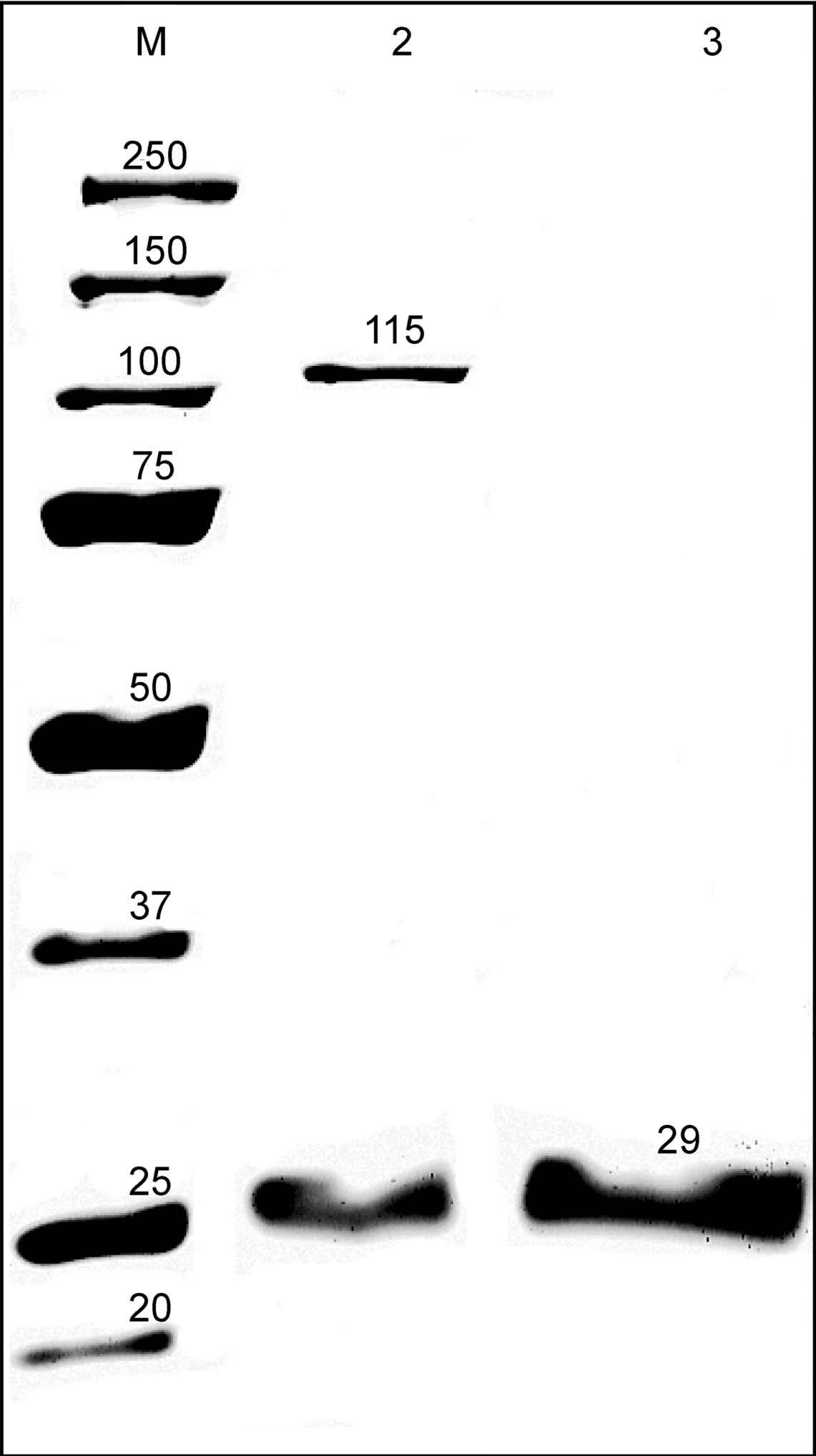

Supplement: Figure S1 — Purification of IlsA. Coomassie-stained 10% SDS-PAGE analysis of the purified GST-IlsA and GST proteins from recombinant E. coli. Lane M, molecular weight markers in KDa. Purified GST-IlsA and GST with apparent molecular weights shown on the gel, was loaded in lane 1 and 2 respectively. (0.51 MB PDF) [file ppat.1000675.s001.pdf]
